# Precision nutrition for type 2 diabetes in Benin: leveraging linear goal programming to optimize diets with emphasis on adequacy, affordability, accessibility, and culture

**DOI:** 10.3389/fnut.2024.1400594

**Published:** 2024-08-08

**Authors:** Halimatou Alaofè, Mahdi Mahdavimanshadi, Carmelle Mizéhoun-Adissoda, Abidemi Okechukwu, Waliou Amoussa Hounkpatin, Edward John Bedrick, Jules Gninkoun, Neng Fan, John Ehiri

**Affiliations:** ^1^Department of Health Promotion Sciences, Mel and Enid Zuckerman College of Public Health, University of Arizona, Tucson, AZ, United States; ^2^Department of Systems and Industrial Engineering, University of Arizona, Tucson, AZ, United States; ^3^School of Nutrition and Dietetics, Faculty of Health Sciences, University of Abomey-Calavi, Cotonou, Benin; ^4^School of Nutrition and Food Science and Technology, Faculty of Agricultural Sciences of the University of Abomey-Calavi, Calavi, Benin; ^5^Department of Epidemiology and Biostatistics, Mel and Enid Zuckerman College of Public Health, University of Arizona, Tucson, AZ, United States; ^6^Faculty of Health Sciences, University of Abomey Calavi, Cotonou, Benin

**Keywords:** adequate diet, acceptable diet, accessible diet, diet costs, linear goal programming, type 2 diabetes, Africa

## Abstract

**Background:**

Nutrition and diet are critical to managing Type 2 diabetes (T2D). Low-income households often face challenges maintaining a healthy and balanced diet due to food insecurity, availability, and cost. To address this issue, we used a linear goal programming (LGP) model to develop nutritionally adequate, affordable, accessible, and culturally acceptable diets for persons with T2D in Benin, a French-speaking sub-Saharan country. The goal was to help persons with T2D manage their condition more effectively.

**Methods:**

We compiled a robust list of local commonly consumed foods in Benin, and calculated their nutritional value using West African food composition tables and food costs per serving from a market survey. Using mathematical optimization techniques, we designed dietary plans that meet the daily nutrient intake recommended by the World Health Organization (WHO) to prevent chronic diseases in normal adults. While adhering to dietary constraints of T2D, we developed optimized diet plans with varying energy levels that meet all nutrient requirements while considering availability, acceptability, and budgetary constraints.

**Results:**

Fifty-two food items and recipes were evaluated to create six low-cost daily menus. Menu 1 was the most affordable at CFA 1,127 (USD 1.88), providing 1890 kcal of energy, while Menu 6 was the most expensive at CFA 1,227 (USD 2.05), providing 1749 kcal. All the menus met the daily WHO minimum requirements for carbohydrates, fat, cholesterol, and fiber content, while other nutrients such as protein, vitamin C, and iron reached the upper limits of the acceptable value range.

**Conclusion:**

Linear goal programming can be an effective tool in helping to obtain optimized adequate, accessible, and culturally acceptable diets at minimal cost by interpreting and translating dietary recommendations into a nutritional model, based on local market prices.

## 1 Introduction

Sub-Saharan Africa (SSA) is currently experiencing a rapid increase in nutrition-related chronic diseases, particularly Type 2 diabetes (T2D), leading to significant health challenges and economic burdens (1, 2). By 2045, T2D rates are expected to rise by 129%, worsening the already high levels of complications and comorbidities and placing immense pressure on the healthcare system (3–6). The Republic of Benin, a French-speaking country in SSA, is also facing a similar trend. Within 7 years, T2D prevalence has surged by 76%, reaching 21.6% in some areas (7). The current healthcare system’s focus on treatment rather than prevention has resulted in a 55.8% increase in diabetes-related disabilities between 2007 and 2017 (8, 9). Moreover, diabetes-related complications have led to alarmingly high mortality rates, estimated at 618 deaths per 100,000 for women and 430 deaths per 100,000 for men (10, 11). Additionally, the population of adults aged 45–65 is expected to rise by 64.8%, contributing to a future increase in T2D cases and healthcare costs (12).

The high rates of morbidity and mortality among persons with T2D in Benin are mainly due to their poor adherence to dietary guidelines (13–15). A healthy diet has proven to effectively improve glycemic control, reduce cardiovascular risks, and prevent complications by lowering body mass index (BMI), hemoglobin A1c (HbA1c), triglycerides, cholesterol levels, and blood pressure (16–18). However, only 20% of persons with T2D in Benin follow the dietary recommendations. Moreover, studies indicate that 20% of these individuals have normal energy intake, 75% have high carbohydrate intake, and 77% have abnormal protein intake (19, 20). In a recent study, limited access to healthy foods, difficulty translating recommendations into practice, lack of information about local foods, and cultural acceptance issues hindered dietary adherence (21). Therefore, providing culturally appropriate and practical dietary recommendations is crucial, considering the health, financial, and cultural challenges faced by those living with T2D in Benin (22, 23).

In this context, linear programming (LP) is a valuable mathematical technique to address Benin’s dietary needs. It involves using an objective function and constraints to find values for decision variables. LP can help identify the most appropriate nutritional changes while also considering constraints such as regional food preferences, serving sizes, and cost (24). This approach has been proven to be effective in creating food-based dietary guidelines, especially for populations facing food security and cost challenges. LP can assess the feasibility of diets with multiple constraints without increasing diet costs, which aligns with nutritional goals for diabetes management (25–30). Its notable advantage lies in using the existing diet as a foundation, thus creating optimal diets with familiar foods. This technique is particularly well-suited to complex African environments where diverse variables and constraints must be considered (24–26).

Although LP has gained popularity in nutrition, most studies focused on optimizing diet plans for healthy and undernourished individuals in SSA (25–30). Previous regional studies used LP to create optimal dietary patterns based on nutrient-based recommendations (31, 32) and evaluate cost constraints (33, 34). These studies address accessibility and adequacy, two of the four A’s of food security (35, 36). In addition, while LP is commonly used in Europe, the United States, and Asia, its application to Benin presents challenges due to the country’s distinctive food intake patterns characterized by high-carbohydrate diets with limited consumption of fruits, vegetables, and whole grains (31, 37–39). Benin’s unique dietary context makes it difficult to directly apply results from studies conducted in other regions. Furthermore, research on dietary patterns in SSA for T2D is lacking. To fill these gaps, this study aims to use linear goal programming (LGP) to develop nutritionally adequate, affordable, accessible, and culturally acceptable diets for persons with T2D. LGP is an extension of LP that addresses multiple objectives. It can provide a more realistic representation of real-world problems than LP, often used to optimize decision-making with independent and dependent variables (40). By optimizing diet plans based on the 4 A’s food security framework (adequacy, acceptability, accessibility, and availability of foods), low-income persons with T2D can improve their dietary adherence and health outcomes.

## 2 Materials and methods

### 2.1 Data preparation

**Figure 1:**
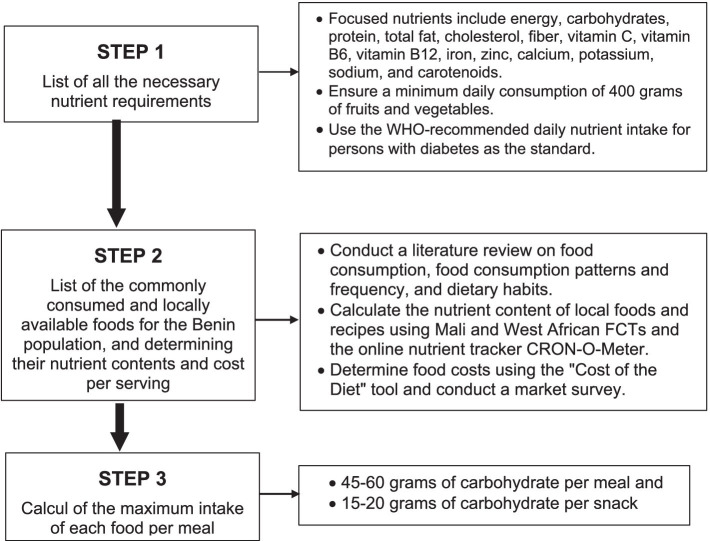
Flow chart describing diet design using linear goal programming.

As a first step in developing affordable, accessible, and culturally acceptable diets that meet nutritional requirements, we followed the data preparation process outlined by Briend et al. (41). The process involved three steps as described in Figure 1: (1) listing all the necessary nutrient requirements, (2) listing the commonly consumed and locally available foods for the Benin population, and determining their nutrient contents and cost per serving, and (3) calculating the maximum intake of each food per meal.

#### 2.1.1 Nutrient requirements

Dietary needs vary according to gender, age, weight, height, and daily activities. We used the World Health Organization’s (WHO) recommended daily nutrient intake for normal adults to prevent chronic diseases, as outlined in Table 1 (42). The nutrients considered were energy, carbohydrates, protein, total fat, cholesterol, fiber, vitamin C, vitamin B6, vitamin B12, iron, zinc, calcium, potassium, sodium, and carotenoids. Total fats include visible fats such as cooking and salad oils, invisible fats found in plant foods like fruits, vegetables, cereals, and nuts, and animal foods such as fish, eggs, meats, and milk products. We also include a minimum daily consumption of 400 grams of fruit and vegetables to promote a balanced and healthy diet (43).

**Table 1:** Recommended daily nutritional intake to prevent chronic diseases.

Dietary factors	Goals (normal nutritional status)
	Man and woman
Energy	1800–2,200 kcal
Carbohydrates*	50–55%
Proteins*	12-15%
Total fat*	< 30%
Cholesterol	< 300 mg/d
Fiber (g)	20–35 g/d
Vitamin B6	>1.3 mg/d
Vitamin B12	>2.4 μg/d
Vitamin C	75–90 mg/d
Vitamin A, Carotenoids	700–900 μg/d
Iron (mg/d)	18–8 mg/d
Zinc (mg/d)	8–11 mg/d
Calcium (mg/d)	1,000 mg/d
Sodium (mg/d)	<1,500 mg/d
Potassium (mg/d)	2,600–3,400 mg/d
Fruits and vegetables	> 400 g

*Percentage of total energy needs.

#### 2.1.2 Foods, nutrient contents, and cost per serving

We created a detailed list of commonly consumed and locally available foods in Benin. To compile this list, we conducted a thorough search of the literature on food consumption, food consumption patterns, dietary habits, and the frequency of food consumption in all three regions (North, Central, and South) of Benin using resources such as Google, Google Scholar, and PubMed. We carefully reviewed all available abstracts and obtained relevant articles. Additionally, we searched for Master’s and doctoral theses related to food consumption in Benin in the Faculty of Agricultural Sciences (FSA) Library of the University of Abomey-Calavi, Benin. In total, we identified 16 studies conducted in the three regions of Benin. Ten of these studies examined food consumption patterns, dietary habits, and food consumption frequency, while the remaining six focused on food consumption through dietary surveys among 21 to 514 participants from different socio-economic statuses in rural and urban areas. Our review also considered national diet surveys conducted to develop Benin food-based dietary guidelines (25) and regional diet surveys to determine adherence to dietary recommendations among persons with T2D (8, 44, 45).

Based on the literature review, including additional information on food frequency patterns, most individuals in Benin ate breakfast, lunch, dinner, and sometimes evening snacks in urban areas (46). With this information, daily nutrient intake was estimated using the foods consumed during these three significant meals and evening snacks. A list of 52 commonly consumed items by the Benin population, both diabetic and non-diabetic, was used to curate food combinations for meal plans. Some foods and recipes were selected based on dietary reference intakes with a low glycemic index, as recommended by the WHO guidelines for persons with T2D (47–49). Fast foods and soft drinks were excluded from the list based on nutritionist observation and feedback. Table 2 lists foods and recipes for different food intervals. The 52 items were classified into foods and recipes for breakfast (30 items), lunch/dinner (36 items), and evening snacks (16 items). The same food items were recommended for lunch and dinner as they could be taken at either meal.

**Table 2:** Commonly consumed foods and recipes used for diet optimization.

Breakfast	Evening snacks	Lunch/dinner
Corn paste; Tomato sauce; Vegetable sauce (Fonman); *Corchorus olitorius* L (crin-crin); Okra (Gombo); Cooked tilapia fish; Fresh cooked chinchard (silivi); Cooked lean lamb; Cooked chicken; White rice, cooked; Fermented corn dough porridge; Sugar; Grilled peanuts; Granulated fermented flour porridge; Fermented corn dough (akassa); Tomato sauce (Moyo); Mixed rice and beans; Boiled chicken eggs; Local cheese; Sweetened wheat doughnut (yovodoko); Bean cake (Akara); Oatmeal; Soja porridge; Evaporated unsweetened milk (peak); Fresh orange; Fresh banana; Fresh papaya; Lemon juice without added sugar; White wheat bread; Cooked beans; Peanut oil; Millet/Sorghum porridge, unsweetened	Fermented cassava (gari); Sugar; Peanut aballs (Klui Klui); Grilled peanuts; Granulated fermented flour porridge; Fermented corn dough porridge; Bean cake; Fresh Orange; Fresh Pineapple; Fresh Banana; Mango; Tea; Bean cake; Boiled chicken eggs; Plain yogurt; Papaya	Corn paste; Tomato sauce; White rice, cooked; Vegetable sauce (Fonman, vernonie, gboman); Amaranth leaves (crin-crin); Cooked tilapia fish; Fresh cooked chinchard (silivi); Cooked lean lamb; Cooked chicken; Yam-couscous (Wassa-wassa); Pepper; Cooked beans; Peanut oil; Palm oil; Local cheese; Fermented corn dough (akassa); Tomato sauce (Moyo); Boiled yam; Pounded yams; Ground nut stew; Cassava-pudding; Beans and rice; Yam dough (Telibô); White wheat bread; Cooked chicken; Boiled cassava; Watermelon; Mango; Pineapple; Avocado; Cassava-pudding (Eba); Pasta; Salad.

Due to the absence of a food composition table (FCT) in Benin, nutrient data for local foods and recipes were sourced from Mali and West African FCTs (50–52) and the online nutrient tracker CRON-O-Meter (53).^1^ However, these sources provided incomplete data, lacking coverage for all commonly consumed local recipes. Consequently, a food consumption survey was conducted in Benin’s three main regions to ascertain these recipes’ ingredients and nutritional values. Given the incomplete information in African food tables, the study focused on energy requirements and 14 essential nutrients for individuals with normal nutritional status, assuming each person would receive 100% of the required nutrients from their diet.

In calculating food costs per serving, this study utilized the “Cost of the Diet” tool and conducted a market survey. The “Cost of the Diet” tool considers factors such as the World Food Composition Database, the World Health Organization’s (WHO) recommended nutrient requirements for various groups, local food prices, and household compositions (54). As staple food prices rose 23.9% in SSA (55), a market survey in the three main regions collected three different prices per food item, including the highest and lowest across all vendors. Raw ingredient weights for cooked composite dishes were measured, and their costs were totaled to determine the overall cost of all ingredients.

#### 2.1.3 Maximum intake of each food per meal

Some researchers and clinicians suggest that adopting a carbohydrate-reduced diet may enhance outcomes for persons with T2D, including improved glycemic control, weight loss, and decreased reliance on hypoglycemic medications. This approach has sparked discussions about the potential effectiveness of treating T2D with a carbohydrate-reduced diet, possibly leading to remission (56, 57). However, most persons with T2D in Benin follow high-carbohydrate diets with low fruits, vegetables, and whole grains (31, 38, 39). To encourage better food choices, maximum amounts of each food type per meal were determined, aligning with Benin’s Food-based dietary guidelines (25), carbohydrate recommendations for diabetics (45–60 grams per meal and 15–20 grams per snack) (58), and expert advice. Portion sizes, calculated in grams and domestic measures, considered the nutritional composition of specific foods and adhered to the nutrition content criteria for one portion of each food group. Table 3 provides examples of foods and their recommended maximum intake per meal. A standard serving size was defined as a 250 mL bowl used to serve corn dough in Benin. A tablespoon (15 mL) and the palm or the thumb were also used as references for portion size.

**Table 3:** Illustrative foods and maximum intake per meal*.

Food item	Weight (g)	Maximum intake per meal
Cereals		
Corn dough, non-fermented	185	1 bowl
Cooked white rice	220	1 bowl
Fermented corn dough (*akassa*)	185	1 bowl
Boiled macaroni	140	1 bowl
White wheat bread	60	1/2 bread
Tubers and roots		
Cooked yam	136	1 bowl
Yam dough (*Telibô*)	175	1 bowl
Fermented cassava (*gari*)	85	1/3 bowl
Vegetables and vegetable sauces		
Tomato sauce	225	1 bowl
Tomato sauce +crincrin	200	1 bowls
Leafy vegetable sauce	160	1 bowl
Beans, nuts and seeds		
Cooked white beans (*white niébé*)	125	1 bowl
Soya porridge, unsweetened	200	1 bowl
Grilled peanuts	19	1/2 bowl
Fruits		
Fresh papaya	95	1 bowl
Fresh banana	75	1 medium
Fresh orange	100	1 medium
Fresh mango	82.5	1 small or 1/2 large
Dairy products		
Evaporated unsweetened milk (*peak*)	85	1/2 milk box
Local cheese	50	The length of two thumbs
Traditional yoghurt	125	1 small container

*Bowl represents 250 mL.

### 2.2 Linear goal programming diet planning model

The LGP diet model was developed to create optimized diet plans for persons with T2D in Benin. These plans aim to meet patients’ energy and nutrient requirements while considering factors such as affordability, cultural acceptance, and local food availability. To achieve this, we used three types of constraints. First, energy and nutrient constraints were implemented based on the WHO’s recommended daily energy and nutrient intake to prevent chronic diseases among normal adults (45, 47–49). Secondly, the maximum portion size for each type of food per meal was determined by relying on the Benin food-based dietary guidelines and low-carbohydrate recommendations for persons with T2D (25, 59). Finally, the model considered acceptability, availability, and cost as constraints by selecting local commonly consumed foods and low-cost diets based on a market survey conducted in the three main regions of the country. The LGP formulation is as follows:


(a) $$\min\;z=\underset{i}{\sum }w_{i}^{u}\left(d_{iu}^{-}+d_{iu}^{+}\right)+w_{i}^{l}\left(d_{il}^{-}+d_{il}^{+}\right)$$

(b) $$\underset{t\in T}{\sum }\underset{m\in M}{\sum }N_{mti}x_{mt}+d_{iu}^{-}-d_{iu}^{+}=UG_{i},\forall i$$

(c) $$\underset{t\in T}{\sum }\underset{m\in M}{\sum }N_{mti}x_{mt}+d_{il}^{-}-d_{il}^{+}=lG_{i},\forall i$$

(d) $$\underset{m\in M}{\sum }x_{mt}=A_{t},\forall t$$

(e) $$d_{iu}^{-},d_{iu}^{+},d_{il}^{-},d_{il}^{+}\geq 0,x_{mt}=\left\{0,1\right\}\forall i,t,m$$

Sets and indices:

i∈I : set of nutrients and energy.

t∈T : set of type meals.

m∈M : set of meals.

Decision variables:

$d_{iu}^{-},d_{iu}^{+}:$ under-achievement and over-achievement of each nutrient and energy ( i ) from upper bound.

$d_{il}^{-},d_{il}^{+}:$ under-achievement and over-achievement of each nutrient and energy ( i ) from lower bound.

$x_{mt}\in \left\{0,1\right\}$ : 1, if the meal ( m ) in type meal ( t ) is selected for menu, otherwise 0.

Parameters:

$w_{i}^{u}$ : The weights for upper bounds in nutrients and energy ( i ).

$w_{i}^{l}$ : The weights for lower bounds in nutrients and energy ( i ).

$N_{mti}$ : Nutrient and energy ( i ) value in meal ( m ) with type meal ( t ).

$A_{t}\in \left\{0,1\right\}$ : 1, if type meal ( t ) should be in menu, otherwise 0.

$UG_{i}$ : Upper bound for nutrient and energy ( i ) in menu.

$lG_{i}$ : Lower bound for nutrient and energy ( i ) in menu.

The objective function (Eq. a) minimizes deviations from nutrient and energy goals. The coefficients assigned to deviational variables in the objective function serve as indicators, reflecting both the significance and desirability of deviations from different nutrient and energy goals. Constraints (Eq. b) and (Eq. c) utilize goal programming constraints to ensure the attainment of specific goals for each nutrient and energy. Constraint (Eq. d) specifies that each type of meal should be either considered or not in the menu. Furthermore, constraint (Eq. e) ensures that decision variables adhere to defined domains.

### 2.3 Data analysis

In this study, the tests were conducted on a MacOS with one Apple M1 chip and 8GB RAM. We solve the LGP proposed using Python 3.10.3 and optimization solver Gurobi 9.1.2.

## 3 Results

Six of the 15 optimized menus generated using LGP had the lowest costs with various energy levels that fell within the lower and upper limits set for kcal (1800–2,200 kcal) as shown in Table 4. Menu 1 had the lowest cost of CFA 1,127 (USD 1.88) with an energy level of 1890 kcal, while Menu 6 has the highest price of CFA 1,227 (2.05 USD) with an energy level of 1749 kcal. Moreover, most of the foods included in the six models are whole grain foods such as millet, sorghum, and maize; vegetables like *C. olitorius* (crin-crin), spinach, and tomato; fruits like oranges, papayas, and lemons; and foods from animal sources such as eggs, fish, and meat. Most of the cooking methods used were steaming, baking, or grilling.

**Table 4:** Six optimal diabetic menus using LGP for adequate calorie intake at minimal cost.

Caloric intake	1890	1,359	1,228
Meal	Model 1	Model 2	Model 3
Breakfast	Cooked beans (1 bowl) Peanut oil (1 tablespoon) Bread (1/2 baguette) Lemon juice (1 bowl) Water (1 cup)	Beans and rice (1 bowl) Tomato stew (1/2 bowl) Grilled fish (56 g) Orange (1 medium) Water (1 cup)	White bread (1/2 baguette) Boiled chicken eggs (2 pieces) Raw vegetables (1/2 bowl) Water (1 cup)
Lunch	Yam flour (1 bowl) Tomato sauce+ crincrin (1 bowl) Grilled fish (56 g) Water (1 cup)	Cooked beans (1 bowl) Peanut oil (1 tablespoon) Pain (1/2 baguette) Water (1 cup)	Wassa wassa (1 bowl) Piment (1/2 bowl) Poisson fume (56 g) Water (1 cup)
Afternoon snack		Tea (+unsweetened sugar)	
Dinner	Vegetables salad with cooked eggs (2 bowls) Bread (1/2 baguette) Water (1 cup)	Macaroni (1 bowl) Tomato sauce (1 bowl) Boiled chicken eggs (2) Water (1 cup)	Akassa (1 bowl) Tomato sauce (1 bowl) Grilled salmon fish (56 g) Water (1 cup)
Total Consumption (kcal/day)	1889.6	1359.4	1228.17
Food cost per day (CFA)	1,127 (USD 1.88)	1,136 (USD 1.90)	1,184 (USD 1.98)

Caloric intake	2034	1,592	1749
Meal	Model 4	Model 5	Model 6
Breakfast	Beans and rice (1 bowl)Tomato stew (1/2 bowl) Grilled fish (56 g) Orange (1 medium) Water (1 cup)	Millet/Sorghum porridge (1.5 bowl) Bean cake, Akara (2 small pieces) Orange (1 medium) Water (1 cup)	Non-fermented corn dough porridge (1.5 bowl) Sweetened wheat doughnut (2 small pieces) Water (1 cup)
Lunch	Cooked beans (1 bowl) Peanut oil (1 tablespoon) Bread (1/2 baguette) Water (1 cup)	Yam flour (1 bowl) Tomato sauce+ crincrin (1 bowl) Grilled fish (56 g) Water (1 cup)	Fermented corn dough, akassa (1 bowl) Tomato sauce, Moyo (1 bowl) Boiled fish (56 g) Papaya (1 medium) Water (1 cup)
Afternoon snack		Grilled peanuts (1/4 bowl)	Orange (1 medium)
Dinner	Rice (1 bowl) Tomato sauce (1 bowl) Grilled Fish (56 g) Water (1 cup)	Akassa (1 bowl) Tomato sauce (1 bowl) Grilled salmon fish (56 g) Water (1 cup)	Rice (1 bowl) Vegetables sauce (1 bowl) Cooked meat (beef) (56 g) Water (1 cup)
Total Consumption (kcal/day)	2033.570	1591.577	1748.940
Food cost per day (CFA)	1,220 (USD 2.04)	1,223 (USD 2.02)	1,227 (USD 2.05)

All six menus also met the constraints’ upper and lower limits, including the recommendations for macronutrients and micronutrients, as displayed in Table 5. The values for carbohydrates, fat, cholesterol, and fiber have reached the constraints’ lower limits. However, other nutrients such as protein, vitamin B12, vitamin C, and iron have reached maximum acceptable levels.

**Table 5:** Nutritional information of the six optimal menus at the lowest cost based on different caloric intake.

		Menu 1: Calorie: 1890	Menu 2: Calorie:1359	Menu 3: Calorie: 1228	Menu 4: Calorie:2034	Menu 5: Calorie:1592	Menu 6: Calorie 1749
	Goals	Consumption	Consumption	Consumption	Consumption	Consumption	Consumption
Carbohydrates (g)	50–55%	173.05 (37%)	88.73 (26%)	97.78 (32%)	229.92 (45%)	92.78 (23%)	166.53 (38%)
Protein (g)	12–15%	75.76 (16%)	46.30 (14%)	72.27 (24%)	65.29 (13%)	50.93 (13%)	49.55 (11%)
Fat (g)	<30%	43.95 (21%)	20.10 (13%)	34.40 (25%)	55.53 (25%)	29.51 (17%)	74.55 (38%)
Cholesterol (mg)	<300 mg/d	161.30	81.96	362.59	155.08	81.96	94.63
Fiber (g)	20–35 g/d	20.20	28.94	26.37	23.56	20.54	22.63
Vitamin A, beta-carotene	>700–900 μg/d	403.13	1349.65	546.98	502.04	1349.65	799.22
Vitamin B6 (mg)	>1.3 mg/d	1.55	0.89	1.20	0.89	0.99	1.22
Vitamin B12	>2.4 μg/d	11.88	17.45	18.68	10.98	17.45	7.72
Vitamin C (mcg)	75–90 mg/d	126.84	126.74	129.75	171.25	126.74	151.78
Iron (mg)	18–8 mg/d	22.23	22.57	12.85	12.25	22.87	24.80
Zinc (mg)	8–11 mg/d	7.73	42.44	5.83	5.92	42.97	7.10
Calcium (mg)	1,000 mg/d	324.66	202.25	223.64	241.69	213.27	255.77
Potassium (mg)	<1,500 mg/d	1365.41	1475.83	1396.20	1540.77	1596.29	1074.30
Sodium (mg)	2,600–3,400 mg/d	1400.27	1516.98	1630.51	1465.39	1518.12	1778.66

**Figure 2:**
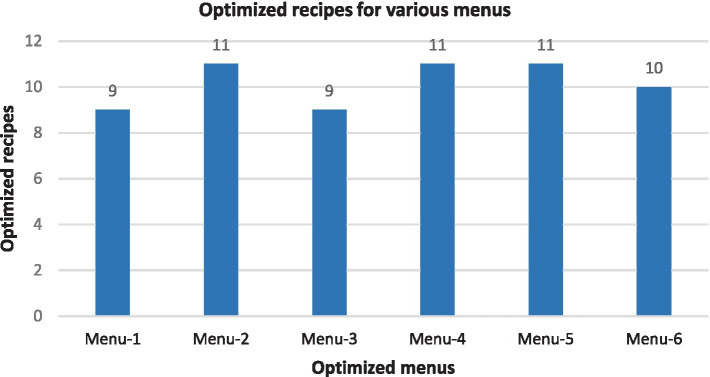
Total number of optimized recipes for the six menus produced.

Finally, out of the 56 recipes commonly consumed and available locally in Benin, six menus had an average of 10 recipes. Menu 1 contained 9 recipes with 1890 kcals of energy. Menu 2 had 11 recipes with 1,359 calories. Menu 3 consisted of 9 recipes with 1,228 Kcals. Menu 4 and Menu 5 had 11 recipes with energy levels of 2034 and 1,592 kcals, respectively. Lastly, menu 6 included 9 recipes with an energy level of 1749 calories, as shown in Figure 2.

## 4 Discussion

Type 2 diabetes (T2D) is increasing in SSA, including Benin, and culturally appropriate and practical dietary recommendations are urgently needed. In the findings, six of 15 optimized menus created through LGP achieved the lowest costs while meeting energy levels between 1800 and 2,200 kcal/day. Specifically, Menu 1 had the lowest price of USD 1.88 at an energy level of 1890 kcal. Despite Models 5 and 6 having lower energy levels, they incurred higher costs of USD 2.02 and USD 2.05, respectively. The six menus predominantly featured whole grains, vegetables, fruits, and animal sources prepared through steaming, baking, or grilling and met upper and lower constraints for nutrients, aligning with dietary recommendations. In addition, the study showcased practicality by compiling an average of 10 recipes per menu from 56 locally available options in Benin, emphasizing diversity in the proposed diabetic menus. Therefore, the study offered feasible solutions to energy and dietary issues by balancing nutritional needs with acceptability, accessibility, and budget limitations. It also suggested that alternative menu combinations could be explored by replacing food recipes with similar nutrient sources in these optimization models. Additionally, food recipes can be adjusted based on diabetes patients’ sugar levels to ensure energy balance (27). As a result, this study assists in managing glucose levels through optimized food menus and encourages a healthy lifestyle for individuals with T2D in Benin or similar environments.

The dietary patterns were modeled based on WHO-recommended daily nutrient intake as constraints. In contrast, other researchers have used more American criteria such as EAR, adequate intake, UL, RDA, or RDI (28, 39, 60–62). Our decision to follow the WHO recommendations was justified because they have been proposed to reduce risk factors for chronic diseases resulting from unhealthy diets and physical inactivity through public health efforts in SSA (42). Additionally, by meeting the higher micronutrient objectives, the modeled diet fulfilled all the requirements for this population, which were low in previous observed amounts (8, 44). Moreover, supplementation with fiber, calcium, iron, vitamin C, and vitamin A would enable individuals to meet their dietary needs if the proposed diet was not adopted. However, individuals should strive to meet their nutrient requirements through healthy eating patterns that include nutrient-dense foods as highlighted in this study. Conversely, unlike studies among persons with T2D in Benin (8, 44), our modeled diets lowered sodium and potassium without increasing the cost. Consequently, the prevalence of high blood pressure and heart disease, two main concerns for diabetes patients, could be reduced (63, 64).

Furthermore, little improvement to the existing diet has been achieved by increasing the consumption of functional foods that effectively treat and prevent diabetes (65). These foods include whole grains (millet and sorghum), rich in fiber; vegetables (crincrin and spinach), rich in fiber, minerals, and vitamins while low in calories; fruits (orange and papaya), packed with fiber, vitamins, and antioxidants; and food from animal sources, rich in proteins. They also contain bioactive compounds that help manage diabetes (66). Moreover, the menus emphasize low glycemic index foods like akassa (GI 18), green vegetable soup (GI 25), akara (GI 43.7), and rice and tomato soup (GI 46.3). Sugary drinks are avoided except for naturally occurring sugar in fruits and vegetables (67–69). Healthy, high-antioxidant drinks such as lemon and orange juice are recommended alternatives. Lean proteins like beans, fish, and peanuts are suggested as protein sources. Further, as per Asmaa et al. (70), the recommended menus stress reducing saturated and trans-fats by utilizing cooking methods like steaming, baking, or grilling, which reduce meal fat content. High intakes of fats and cholesterol have been associated with a higher risk of CVD and diabetes, so lowering this could also improve their health (66–68). However, communication with health workers and dietitians will be essential to promote healthier alternatives to individuals while making them more available (increased physical and economic access) in community stores and markets.

We aim to produce an adequate, accessible, and culturally acceptable diet that helps to manage T2D so that it is affordable and achievable for low-income individuals. In the study, energy levels were categorized into three ranges: 1200–1,600 kcal per day, 1,600–1800 kcal per day, and 1800–2,200 kcal per day, ideal for overweight or obese patients. Managing T2D in SSA is challenging due to individuals being overweight at diagnosis and gaining weight during treatment (2, 65, 71–73). Dietary plans should focus on reducing energy intake while ensuring optimal nutrient consumption. The carbohydrate values are now at the lowest limit. Previous studies have shown that very low-carbohydrate diets (20% or less of total calories) and low-carbohydrate diets (40% or less of total calories) can improve outcomes for T2D, including weight loss, better glycemic control, and reduced use of hypoglycemic medications (74–76). In addition, while budget constraints remain a concern because of food price rises, our optimized diets maintain a minimum cost within a specific range that, in this study, is below USD 2.05 each day, including three or four main meals. However, we did not apply a cost constraint to our models since previous research has shown that it can adversely affect diet nutrient density and food selection (24). We selected commonly consumed and easily accessible foods. Identifying affordable food patterns that provide the necessary nutrients can help reduce social inequalities, such as those in Benin. However, it is also critical to address the underlying causes of food insecurity and dietary inadequacy in the country.

However, this study had some limitations. The selection of optimal food recipes is limited to T2D patients without significant complications. However, optimization models can be used to extend this study to patients with other comorbidities, such as gestational diabetes, hypertension, cardiovascular disease, and other chronic illnesses. Moreover, we assumed that all food prices were based on purchases made at markets or street vendors without considering cooking costs or removing non-edible portions. The diet cost may have been underestimated for home-prepared food bought as ready-to-eat meals, and conversely, ready-to-eat food prepared at home may have been overestimated. While acknowledging the study’s limitations, it is notable for its comprehensive approach, targeting urban and rural populations across the three main regions of Benin. The selected foods for optimization are readily available, accessible, and culturally acceptable, rendering the food intake data representative of the general population. Moreover, the study employs domestic measurements to convey servings in easily understandable amounts, potentially improving compliance with recommended portion sizes and numbers (71). Furthermore, adding locally cooked recipes with food composition data from a survey in Benin’s three main regions strengthens the study’s foundation. Our next steps are to evaluate the proposed diets among 30 people with T2D after appropriate training. We will create user-friendly guidance for using the menus and address other lifestyle factors. Evaluation will include pre- and post-diet measurements of weight and HbA1c.

## 5 Conclusion

This study underscores the potential of LGP as an effective tool for crafting affordable, culturally acceptable, accessible, and nutritionally adequate diets tailored to the needs of individuals with T2D in Benin, a Francophone SSA country. Leveraging local market prices, this method transforms dietary recommendations into an optimized nutritional model at minimal cost. The focus on selecting food recipes with low carbohydrate and fat content while meeting all essential nutrients for diabetes patients holds crucial significance for menu development. By identifying widely available food recipes and alleviating the economic burden on Benin’s population, this research provides a valuable resource for T2D patients, offering an ideal menu to sustain a healthy lifestyle at a reduced cost.

## Data availability statement

The data that support the findings of this study are available on request from the corresponding author.

## Author contributions

HA: Conceptualization, Data curation, Formal analysis, Funding acquisition, Investigation, Methodology, Project administration, Resources, Software, Validation, Writing – original draft, Writing – review & editing. MM: Data curation, Formal analysis, Methodology, Software, Writing – original draft, Writing – review & editing. CM-A: Conceptualization, Methodology, Writing – original draft, Writing – review & editing. AO: Conceptualization, Methodology, Writing – original draft, Writing – review & editing. WAH: Conceptualization, Formal analysis, Funding acquisition, Investigation, Methodology, Supervision, Validation, Writing – original draft, Writing – review & editing. EB: Conceptualization, Funding acquisition, Methodology, Supervision, Validation, Writing – original draft, Writing – review & editing. JG: Conceptualization, Methodology, Writing – original draft, Writing – review & editing. NF: Conceptualization, Methodology, Writing – original draft, Writing – review & editing. JE: Conceptualization, Funding acquisition, Methodology, Project administration, Supervision, Validation, Writing – original draft, Writing – review & editing.

## Footnotes

^1^https://cronometer.com/

## References

[1] Motala AA Mbanya JC Ramaiya K Pirie FJ Ekoru K. Type 2 diabetes mellitus in sub-Saharan Africa: challenges and opportunities. Nat Rev Endocrinol. (2022) 18:219–29. doi: 10.1038/s41574-021-00613-y, PMID: 34983969

[2] Goedecke JH Mendham AE. Pathophysiology of type 2 diabetes in sub-Saharan Africans. Diabetologia. (2022) 65:1967–80. doi: 10.1007/s00125-022-05795-2, PMID: 36166072 PMC9630207

[3] International Diabetes Federation. IDF Diabetes Atlas. 9th ed. Brussels, Belgium: International Diabetes Federation (2019).

[4] Saeedi P Petersohn I Salpea P Malanda B Karuranga S Unwin N. Global and regional diabetes prevalence estimates for 2019 and projections for 2030 and 2045: results from the international diabetes federation diabetes atlas, 9th edition. Diabetes Res Clin Pract. (2019) 157:107843. doi: 10.1016/j.diabres.2019.107843 31518657

[5] Khan MAB Hashim MJ King JK Govender RD Mustafa H Al KJ. Epidemiology of type 2 diabetes - global burden of disease and forecasted trends. J Epidemiol Glob Health. (2020) 10:107–11. doi: 10.2991/jegh.k.191028.001, PMID: 32175717 PMC7310804

[6] Pastakia SD Pekny CR Manyara SM Fischer L. Diabetes in sub-Saharan Africa - from policy to practice to progress: targeting the existing gaps for future care for diabetes. Diabetes Metab Syndr Obes. (2017) 10:247–63. doi: 10.2147/DMSO.S126314, PMID: 28790858 PMC5489055

[7] WHO. WHO STEPwise approach to surveillance (STEPS). Benin. Geneva: WHO (2015).

[8] Houngla MFN. Pratiques alimentaires et gestion du diabète chez les diabétiques suivis au Centre National Hospitalier et Universitaire et à la Banque d’insuline de Cotonou au Bénin. Montreal (Canada): University of Montreal (2020).

[9] Adeya G Bigirimana A Cavanaugh K Franco L. *Rapid assessment of the health system in Benin*. (2006).

[10] Institute for Health Metrics and Evaluation. *Benin*. (2017). Available at: http://www.healthdata.org/benin (Accessed February 15, 2024).

[11] GBD. Causes of death collaborators. Global, regional, and national age-sex-specific mortality for 282 causes of death in 195 countries and territories, 1980-2017: a systematic analysis for the global burden of disease study 2017. Lancet (2018). (2017) 392:1736–88. doi: 10.1016/S0140-6736(18)32203-7 PMC6227606 30496103

[12] Ministry of Health (Benin). Benin Health Statistical Yearbook. Porto-Novo, Benin: Ministry of Health (Benin) (2021).

[13] D’Souza MS Karkada SN Parahoo K Venkatesaperumal R Achora S Cayaban AR. Self-efficacy and self-care behaviours among adults with type 2 diabetes. Appl Nurs Res. (2017) 36:25–32. doi: 10.1016/j.apnr.2017.05.004 28720235

[14] Raj GD Hashemi Z Soria Contreras DC Babwik S Maxwell D Bell RC. Adherence to diabetes dietary guidelines assessed using a validated questionnaire predicts glucose control in adults with type 2 diabetes. Can J Diabetes. (2018) 42:78–87. doi: 10.1016/j.jcjd.2017.04.006, PMID: 28648765

[15] Al-Salmi N Cook P D'Souza MS. Diet adherence among adults with type 2 diabetes mellitus: a concept analysis. Oman Med J. (2022) 37:e361. doi: 10.5001/omj.2021.69, PMID: 35441038 PMC8994850

[16] Asaad G Soria-Contreras DC Bell RC Chan CB. Effectiveness of a lifestyle intervention in patients with type 2 diabetes: the physical activity and nutrition for diabetes in Alberta (PANDA) trial. Healthcare (Basel). (2016) 4:73. doi: 10.3390/healthcare4040073, PMID: 27690122 PMC5198115

[17] Milenkovic T Bozhinovska N Macut D Bjekic-Macut J Rahelic D Velija Asimi Z. Mediterranean diet and type 2 diabetes mellitus: a perpetual inspiration for the scientific world. A review. Nutrients. (2021) 13:1307. doi: 10.3390/nu13041307, PMID: 33920947 PMC8071242

[18] Antoniotti V Spadaccini D Ricotti R Carrera D Savastio S Goncalves Correia FP. Adherence to the Mediterranean diet is associated with better metabolic features in youths with type 1 diabetes. Nutrients. (2022) 14:596. doi: 10.3390/nu14030596, PMID: 35276957 PMC8840273

[19] Mathews E Thomas E Absetz P D’Esposito F Aziz Z Balachandran S. Cultural adaptation of a peer-led lifestyle intervention program for diabetes prevention in India: the Kerala diabetes prevention program (K-DPP). BMC Public Health. (2018) 17:974. doi: 10.1186/s12889-017-4986-0, PMID: 29298703 PMC6389141

[20] Godman B Basu D Pillay Y Mwita JC Rwegerera GM Anand Paramadhas BD. Review of ongoing activities and challenges to improve the Care of Patients with Type 2 diabetes across Africa and the implications for the future. Front Pharmacol. (2020) 11:108. doi: 10.3389/fphar.2020.00108, PMID: 32265688 PMC7098994

[21] Alaofè H Yeo S Okechukwu A Magrath P Amoussa Hounkpatin W Ehiri J. Cultural considerations for the adaptation of a diabetes self-management education program in Cotonou, Benin: lessons learned from a qualitative study. Int J Environ Res Public Health. (2021) 18:8376. doi: 10.3390/ijerph18168376, PMID: 34444125 PMC8393923

[22] Alassani A Dovonou C Gninkoun J Wanvoegbe A Attinsounon C Codjo L. Perceptions and practices of people with diabetes mellitus at the Centre National University Hospital Hubert Maga Koutoucou Cotonou. Le Mali Med. (2017) 32:23–7. PMID: 30079690

[23] Wanvoegbe FA Agbodande KA Alassani A Aviansou A Gninkoun J Amoussou-Guenou D. Evaluation de l’observance thérapeutique chez les diabétiques au Bénin. Med Afr Noire. (2018) 65:7.

[24] van Dooren C. A review of the use of linear programming to optimize diets, nutritiously, economically and environmentally. Front Nutr. (2018) 21, 5:48. doi: 10.3389/fnut.2018.00048 PMC6021504 29977894

[25] Levesque S Delisle H Agueh V. Contribution to the development of a food guide in Benin: linear programming for the optimization of local diets. Public Health Nutr. (2015) 18:622–31. doi: 10.1017/S1368980014000706, PMID: 24762926 PMC10271684

[26] Buttriss JL Briend A Darmon N Ferguson EL Maillot M Lluch A. Diet modelling: how it can inform the development of dietary recommendations and public health policy. Nutr Bull. (2014) 39:115–25. doi: 10.1111/nbu.12076

[27] Paidipati KK Komaragiri H Chesneau C. Pre-Emptive and non-pre-Emptive goal programming problems for optimal menu planning in diet Management of Indian Diabetes Mellitus Patients. Int J Environ Res Public Health. (2021) 18:842. doi: 10.3390/ijerph18157842 34360135 PMC8345798

[28] Johnson-Down L Willows N Kenny TA Ing A Fediuk K Sadik T. Optimization modeling to improve the diets of first nations individuals. J Nutr Sci. (2019) 8:e31. doi: 10.1017/jns.2019.30, PMID: 31595187 PMC6764187

[29] Verly-Jr E Pereira ADS Marques ES Horta PM Canella DS Cunha DB. Reducing ultra-processed foods and increasing diet quality in affordable and culturally acceptable diets: a study case from Brazil using linear programming. Br J Nutr. (2021) 126:572–81. doi: 10.1017/S0007114520004365, PMID: 33143759

[30] Rajikan R Zaidi N Elias SM Shahar S Abdul Manaf Z Mohd YN. Construction of healthy and palatable diet for low socioeconomic female adults using linear programming. Int J Adv Sci Eng Inf Technol. (2017) 7:125. doi: 10.18517/IJASEIT.7.1.1191

[31] Okubo H Sasaki S Murakami K Yokoyama T Hirota N Notsu A. Designing optimal food intake patterns to achieve nutritional goals for Japanese adults through the use of linear programming optimization models. Nutr J. (2015) 14:57. doi: 10.1186/s12937-015-0047-7, PMID: 26048405 PMC4470056

[32] Ferguson EL Darmon N Fahmida U Fitriyanti S Harper TB Premachandra IM. Design of optimal food-based complementary feeding recommendations and identification of key ‘problem nutrients’ using goal programming. J Nutr. (2006) 136:2399–404. doi: 10.1093/jn/136.9.2399, PMID: 16920861

[33] Darmon N Ferguson EL. Briend a impact of a cost constraint on nutritionally adequate food choices for French women: an analysis by linear programming. J Nutr Educ Behav. (2006) 38:82–90. doi: 10.1016/j.jneb.2005.11.028, PMID: 16595286

[34] Maillot M Darmon N Drewnowski A. Are the lowest-cost healthful food plans culturally and socially acceptable? Public Health Nutr. (2010) 13:1178–85. doi: 10.1017/S1368980009993028, PMID: 20105388 PMC4103898

[35] Guiné RPF Pato MLJ Costa CAD Costa DVT Silva PB Martinho VJP. Food security and sustainability: discussing the four pillars to encompass other dimensions. Food Secur. (2021) 10:732. doi: 10.3390/foods10112732 PMC8622412 34829013

[36] Calloway EE Carpenter LR Gargano T Sharp JL Yaroch AL. New measures to assess the “other” three pillars of food security–availability, utilization, and stability. Int J Behav Nutr Phys Act. (2023) 20:51. doi: 10.1186/s12966-023-01451-z, PMID: 37101157 PMC10134599

[37] Perignon M Masset G Ferrari G Barré T Vieux F Maillot M. How low can dietary greenhouse gas emissions be reduced without impairing nutritional adequacy, affordability and acceptability of the diet? A modelling study to guide sustainable food choices. Public Health Nutr. (2016) 19:2662–74. doi: 10.1017/S1368980016000653 27049598 PMC10448381

[38] Cleveland LE Escobar AJ Lutz SM Welsh SO. Method for identifying differences between existing food intake patterns and patterns that meet nutrition recommendations. J Am Diet Assoc. (1993) 93:556–63. doi: 10.1016/0002-8223(93)91816-9, PMID: 8315166

[39] Barré T Vieux F Perignon M Cravedi JP Amiot MJ Micard V. Reaching nutritional adequacy does not necessarily increase exposure tonfood contaminants: evidence from a whole-diet modeling approach. J Nutr. (2016) 146:2149–57. doi: 10.3945/jn.116.234294, PMID: 27629574

[40] Orumie U Ebong D. A glorious literature on linear goal programming algorithms. Am J Oper Res. (2014) 4:59–71. doi: 10.4236/ajor.2014.42007

[41] Briend N Darmon EF Erhardt JG. Linear programming: a mathematical tool for analyzing and optimizing children’s diets during the complementary feeding period. J Pediatr Gastroenterol Nutr. (2003) 36:12–22. doi: 10.1097/00005176-200301000-00006 12499991

[42] World Health Organization. Global strategy on diet, physical activity and health. Geneva: World Health Organization (2004).

[43] Mooradian AD Failla M Hoogwerf B Maryniuk M Wylie-Rosett J. Selected vitamins and minerals in diabetes. Diabetes Care. (1994) 17:464–79. doi: 10.2337/diacare.17.5.464 8062625

[44] Gbedan M Adepoju O Amoussa H Djrolo F. *Portion Sizes, Dietary Adequacy Of Diabetic Diets, Nutritional Status And Glycaemic Control Among Type 2 Out-Patients At CNHU in Cotonou, Benin Republic*. Abomey-Calavi (Benin): University of Abomey-Calavi. (2019).

[45] Ategbo EAD. Food and nutrition insecurity in northern Benin: Impact on growth performance of children and on year to year nutritional status of adults. Wageningen: Landbouwuniversiteit Wageningen (1993). 150 p.

[46] Ministère de la Prospective. Etude sur les Normes de Consommation des Principaux Produits Vivriers et de L’amélioration des Conditions de Vie au Bénin; CePED. Paris, France: Du Développement de L’évaluation des Politiques Publiques et de la Coordination de L’action Gouvernementale; Centre de Partenariat et D’expertise pour le Développement Durable (2010).

[47] Wheeler ML Dunbar SA Jaacks LM Karmally W Mayer-Davis EJ Wylie-Rosett J. Macronutrients, food groups, and eating patterns in the management of diabetes: a systematic review of the literature. Diabetes Care. (2012) 35:434–45. doi: 10.2337/dc11-2216, PMID: 22275443 PMC3263899

[48] Brand-Miller J Hayne S Petocz P Colagiuri S. Low–glycemic index diets in the management of diabetes: a meta-analysis of randomized controlled trials. Diabetes Care. (2003) 26:2261–7. doi: 10.2337/diacare.26.8.2261 12882846

[49] Jenkins DJ Wolever TM Buckley G Lam KY Giudici S Kalmusky J. Low-glycemic-index starchy foods in the diabetic diet. Am J Clin Nutr. (1988) 48:248–54. doi: 10.1093/ajcn/48.2.248, PMID: 3407604

[50] Calloway DH Murphy S Bunch S Woerner J. *World food 2 dietary assessment system*. (1994). Available at: http://www.fao.org/infoods (Accessed January 28, 2024).

[51] Nordeide M. Table de composition d’aliments du Mali. Oslo: Institut de Nutrition (1998).

[52] Stadlmayr B Charrondiere U Enujiugha V Bayili RG Fagbohoun EG Samb B. Table de composition des aliments d’Afrique de l’Ouest. Rome: FAO (2012).

[53] CRON-O-Meter. (2023). Available at: https://cronometer.com/ (Accessed September 24, 2023).

[54] Save the Children UK. *The cost of the diet–A practitioner’s guide*. (2023). Available at: http://www.savethechildren.org.uk/en/54_9288.htm (Accessed March 2, 2024).

[55] Unsal F Spray J Okou C. Staple food prices in sub-Saharan Africa: an empirical assessment. Washington, DC: International Monetary Fund Papers (2022). 1 p.

[56] Feinman RD Pogozelski WK Astrup A Bernstein RK Fine EJ Westman EC. Dietary carbohydrate restriction as the first approach in diabetes management: critical review and evidence base. Nutrition. (2015) 31:1–13. doi: 10.1016/j.nut.2014.06.011, PMID: 25287761

[57] Saslow LR Summers C Aikens JE Unwin DJ. Outcomes of a digitally delivered low-carbohydrate type 2 diabetes self-management program: 1-year results of a single-arm longitudinal study. JMIR Diabetes. (2018) 3:e12. doi: 10.2196/diabetes.9333, PMID: 30291081 PMC6238840

[58] Gray A Threlkeld RJ. Nutritional recommendations for individuals with diabetes In: Feingold KR Anawalt B Blackman MR Boyce A Chrousos G Corpas E, editors. Endotext. South Dartmouth, MA: MDText.com, Inc. (2000)

[59] Anderson A Barton K Craigie A Freeman J Gregor A Stead M. Exploration of adult food portion size tools. Edinburgh: NHS Health Scotland (2008).

[60] Brimblecombe J Ferguson M Liberato SC O'Dea K Riley M. Optimisation modelling to assess cost of dietary improvement in remote aboriginal Australia. PLoS One. (2013) 8:e83587. doi: 10.1371/journal.pone.0083587, PMID: 24391790 PMC3877064

[61] Marty L Dubois C Gaubard MS Maidon A Lesturgeon A Gaigi H. Higher nutritional quality at no additional cost among low-income households: insights from food purchases of ‘positive deviants’. Am J Clin Nutr. (2015) 102:190–8. doi: 10.3945/ajcn.114.104380, PMID: 26016868

[62] Lluch A Maillot M Gazan R Vieux F Delaere F Vaudaine S. Individual diet modeling shows how to balance the diet of French adults with or without excessive free sugar intakes. Nutrients. (2017) 9:E162. doi: 10.3390/nu9020162 PMC5331593 28230722

[63] Zhang Z Cogswell ME Gillespie C Fang J Loustalot F Dai S. Association between usual sodium and potassium intake and blood pressure and hypertension among U.S. adults: NHANES 2005–2010. PLoS One. (2013) 8:e75289. doi: 10.1371/journal.pone.0075289 24130700 PMC3794974

[64] D’Elia L Rossi G Ippolito R Cappuccio FP Strazzullo P. Habitual salt intake and risk of gastric cancer: a meta-analysis of prospective studies. Clin Nutr. (2012) 31:489–98. doi: 10.1016/j.clnu.2012.01.003, PMID: 22296873

[65] Roberfroid MB. Inulin-type fructans: functional food ingredients. J Nutr. (2007) 137:2493S–502S. doi: 10.1093/jn/137.11.2493S, PMID: 17951492

[66] Oniang'o RK Mutuku JM Malaba SJ. Contemporary African food habits and their nutritional and health implications. Asia Pac J Clin Nutr. (2003) 12:231–6. 14505997

[67] Mbanya JC Mfopou JK Sobngwi E Mbanya DN Ngogang JY Cameroon Study. Metabolic and hormonal effects of five common African diets eaten as mixed meals: the Cameroon study. Eur J Clin Nutr. (2003) 57:580–5. doi: 10.1038/sj.ejcn.1601592 12700620

[68] Serwaa Yeboah E Agbenohervi JK Owiah SG. Glycemic index of five Ghanaian corn and cassava staples. J Food Nutr Res. (2019) 7:624–31. doi: 10.12691/jfnr-7-9-1

[69] Rosane M Wilfred N. Functional foods of sub-Saharan Africa and their implications in the management of type 2 diabetes: a review. Food Sci Nutr. (2023) 12:24–34. doi: 10.1002/fsn3.3764, PMID: 38268906 PMC10804129

[70] Asmaa A Zzaman W Tajul A. Effect of superheated steam cooking on fat and fatty acid composition of chicken sausage. Int Food Res J. (2015) 22:598–605.

[71] Alaofè H Hounkpatin WA Djrolo F Ehiri J Rosales C. Knowledge, attitude, practice and associated factors among patients with type 2 diabetes in Cotonou, southern Benin. BMC Public Health. (2021) 21:339. doi: 10.1186/s12889-021-10289-8, PMID: 33579243 PMC7881446

[72] Duhuze Karera MG Wentzel A Ishimwe MCS Gatete JD Jagannathan R Horlyck-Romanovsky MF. A scoping review of trials designed to achieve remission of type 2 diabetes with lifestyle intervention alone: implications for sub-Saharan Africa. Diabetes Metab Syndr Obes. (2023) 16:677–92. doi: 10.2147/DMSO.S403054, PMID: 36923683 PMC10010137

[73] Oza-Frank R Cheng YJ Narayan KMV Gregg EW. Trends in nutrient intake among adults with diabetes in the United States: 1988-2004. J Am Diet Assoc. (2009) 109:1173–8. doi: 10.1016/j.jada.2009.04.007, PMID: 19559133

[74] Hussain TA Mathew TC Dashti AA Asfar S Al-Zaid N Dashti HM. Effect of low-calorie versus low-carbohydrate ketogenic diet in type 2 diabetes. Nutrition. (2012) 28:1016–21. doi: 10.1016/j.nut.2012.01.016, PMID: 22673594

[75] Yancy W Foy M Chalecki A Vernon M Westman E. A low-carbohydrate, ketogenic diet to treat type 2 diabetes. Nutr Metab. (2005) 2:34. doi: 10.1186/1743-7075-2-34, PMID: 16318637 PMC1325029

[76] Reaven GM. Effect of dietary carbohydrate on the metabolism of patients with non-insulin dependent diabetes mellitus. Nutr Rev. (1986) 44:65–73. doi: 10.1111/j.1753-4887.1986.tb07589.x, PMID: 3703391

